# Eating Disorder and Autism Collaborative project outline: promoting eating disorder research embedded in a neurodiversity-affirming culture

**DOI:** 10.1192/bjb.2024.61

**Published:** 2025-08

**Authors:** Fiona Duffy, Karri Gillespie-Smith, Helen Sharpe, Kyle Buchan, Emy Nimbley, Ellen Maloney, Michelle Sader, Sarah Kettley, Jess Kerr-Gaffney, Gordon Waiter, Kate Tchanturia

**Affiliations:** 1 University of Edinburgh, UK; 2 University of Aberdeen, UK; 3 Edinburgh College of Art, UK; 4 King's College London, UK; 5 South London and Maudsley NHS Trust, London, UK

**Keywords:** Eating disorders, autism, participatory research, neurodiversity

## Abstract

EDAC (Eating Disorders and Autism Collaborative) is an innovative project aiming to increase research capacity by supporting collaboration in the fields of eating disorders and autism. EDAC comprises four integrated workstreams to co-produce interdisciplinary research, directed by Autistic individuals with lived experience of eating disorders. Workstream 1 will outline best collaborative practices, informing the research network. Workstream 2 will use arts-based methodologies to set research priorities, with emphasis on the perspectives of underrepresented groups. Workstream 3 will support interdisciplinary collaborations to develop innovative research. Finally, workstream 4 will maximise knowledge mobilisation with the aim of reducing barriers to rapid incorporation of research into policy and clinical practice. A core aim of EDAC is to embed a neurodiversity-affirming culture within eating disorder research and to support the development of a new generation of researchers conducting innovative and meaningful research with the potential to improve clinical outcomes.

There is a lack of visibility of research on eating disorders in comparison with other mental health conditions.^1^ This is partly due to historical underfunding, with eating disorder research receiving just 1% of UK mental health research funding per year between 2015 and 2019.^2^ It may also reflect the marginalisation of eating disorder research owing to stigmatisation and a perception of eating disorders as being less severe or important than other mental health difficulties.^1,3^ Eating disorder research is mainly published in specialist eating disorder journals; Solmi and colleagues^1^ reported that of all the papers on eating disorders published in 2018, only 0.2% were published in the top psychiatry journals, despite eating disorders affecting up to 5% of the population at any one time. As a result, and despite eating disorders having a strong overlap with other conditions and neurotypes, eating disorder research lacks visibility and is carried out in isolation from other research fields. Schmidt and colleagues^3^ highlighted a lack of UK strategy for eating disorder research and identified priority areas for this field. These included a need for structural support for training and retention of the next generation of eating disorder researchers. The main aim of EDAC (Eating Disorders and Autism Collaborative) is therefore to increase eating disorder research innovation and capacity by supporting collaborations across diverse groupings of disciplines, led and directed by individuals with living/lived experience, with a spotlight on research in eating disorders in Autistic individuals.

Between 20 and 30% of individuals with anorexia nervosa display diagnostic features characteristic of autism,^4^ and, conversely, in the Autistic population, around 27% display eating disorder symptoms.^5^ Individuals who report more autistic features present more severe eating disorder psychopathology^6,7^ and longer illness duration,^8^ are more likely to require in-patient treatment or nasogastric feeding^9,10^ and report poorer global outcomes post treatment.^11,12^ In addition, concerns have been raised about the effectiveness and potential harms of traditional eating disorder treatments for Autistic populations.^13,14^ This has led to calls to move away from adaptions of traditional, neurotypical-based eating disorder assessments and treatments and develop new approaches embedded within a neurodiversity-affirming framework.^15^

Currently there is a lack of high-quality evidence in this area, leading to the recent Scottish Intercollegiate Guidelines Network^16^ eating disorder committee being unable to make clinical recommendations for Autistic individuals with eating disorders, a position also mirrored by the National Institute of Health and Care Excellence.^17^ Meanwhile, eating disorder clinicians have expressed a lack of confidence in treating Autistic individuals with eating disorders.^18^ To inform eating disorder assessment and clinical intervention in Autistic individuals, research is required to better understand the living/lived experience of co-occurring autism and eating disorders.^19^ To support this, we need to develop an interdisciplinary understanding of the Autistic experience of eating disorders, as well as learning what makes some Autistic individuals more vulnerable to eating disorders, the mechanisms that underpin this and targets for associated interventions.

## EDAC

The EDAC project is jointly funded by the Medical Research Council (MRC), the Economic and Social Research Council (ESRC), the Arts and Humanities Research Council (AHRC), the National Institute for Health and Care Research and the Medical Research Foundation as part of the ‘New collaborations to support eating disorders research’ programme. The aim of this joint funding commitment is to initiate novel interdisciplinary collaborations in eating disorder research and increase capacity in the field. The object of the funding is to ‘enhance knowledge and understanding of the biological, psychological and social causes of eating disorders, to help improve treatments and prevention strategies’.^20^ The research call had a particular focus on increasing collaborations between scientists in eating disorders, as well as involving researchers in other fields, and for projects to demonstrate a strong commitment to involving people with living/lived experience of eating disorders.

This paper provides an overview of EDAC, identifying the goals, tasks and timelines of the project, and is intended to support both planning and communication. This is distinct from a research protocol, which comprises a detailed description of an individual study's research design and methodology; we anticipate that a minimum of six EDAC research protocols, aligned with different workstreams, will be published. EDAC comprises four integrated workstreams over 2 years that will be used to support the creation of an interdisciplinary network to carry out collaborative and living/lived experience-led research in eating disorders and autism. Workstream 1 will form a basis for all future research in the network, bringing together the community to outline best collaborative practices and ethical issues that will inform all work aligned with the research network. Workstream 2 will use arts-based methodologies to set research priorities, with an emphasis on capturing the perspectives of underrepresented groups. Workstream 3 will showcase interdisciplinary proof-of-concept work with the aim of pump-priming collaborative working in the key areas of autism and eating disorders. Finally, workstream 4 will maximise knowledge mobilisation by hosting a policy–clinical think tank event to identify barriers and facilitators to incorporating research into practice, and innovative clinical work via the Pathway for Eating Disorders and Autism developed from Clinical Experience (PEACE; www.peacepathway.org).

In addition to this interdisciplinary research activity and associated publications, we anticipate developing several key outputs, including best practice guidance in ethical co-production of research in eating disorders and autism; an arts-based exhibition to promote greater understanding of the experience of eating disorders for Autistic individuals; and a tailored clinical assessment toolkit for specialist eating disorder services.

## Research culture

There has been a significant sociocultural shift in discourse and understanding regarding autism research, theory and practice. The neurodiversity movement is a recent social view, emerging from the broader civil rights and disability rights movements, that opposes traditional medicalised approaches that conceptualise autism as a disorder that needs to be fixed or cured.^21^ This neurodiversity movement advocates that neurological development and function vary across all humans and is a fundamental concept of diversity.^22^ Within this framework, autism is considered a different ‘neurotype’ as opposed to a distinct condition reflective of underlying deficits or abnormalities. Importantly, it should be emphasised that within neurodiverse-affirming approaches, such differences can confer both advantages and disadvantages, depending on the circumstances.^23^

Neurodivergent experiences and perceptions within a society that adheres more to neurotypical frameworks have been proposed to contribute to historical misconceptions around communication and interaction in Autistic people.^24^ Therefore, it is imperative that researchers begin applying a neurodivergence lens to theory, research and interventions to better capture neurodiverse conceptualisations of clinical conditions such as eating disorders, informing further neurodiversity affirming research, clinical assessments and interventions. Applying a neurodivergence lens to research is facilitated by co-production (working on research together without privileging one type of knowledge over another) with the Autistic community. Within EDAC, we will actively explore Autistic perspectives and experiences of eating disorders using qualitative and art-based methodologies to guide further research. Co-production will be used to set research priorities for Autistic individuals with eating disorders, and the project will provide the Autistic community with research skills to support career development, employability and the promotion of Autistic researchers within the workforce. By embedding a neurodiverse-affirming culture within research and actively supporting and promoting Autistic researchers, future autism and eating disorder research may hope to generate more inclusive, meaningful and translatable research.

## Workstreams

**Fig. 1:**
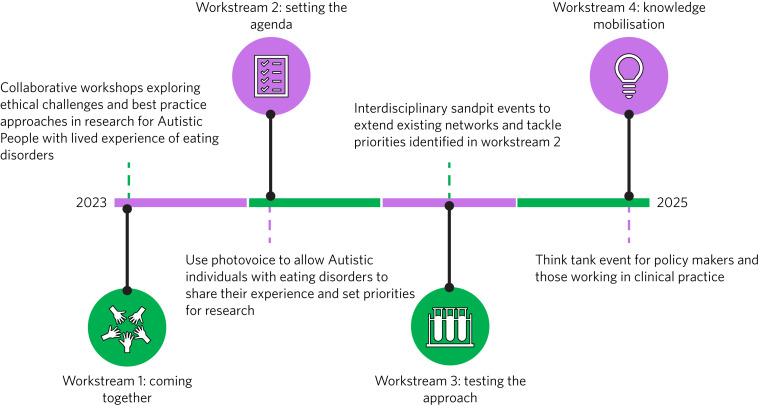
Eating Disorders and Autism Collaborative research network workstreams.

EDAC comprises four integrated workstreams that will take place over a 2-year period (Fig. 1). Workstream 1 will take place between August 2023 and January 2024. It will bring together the community to outline best practice guidelines on ethical co-production, which will inform workstreams 2–4 and all research aligned with the EDAC network. Workstream 2 will take place between November 2023 and August 2024 using arts-based methodologies to set research priorities. These priorities will then be used to select interdisciplinary proof-of-concept proposals in workstream 3, which will occur between May 2024 and May 2025 and will also be underpinned by the expectations of co-production set out in workstream 1. Finally, workstream 4 (August 2024 to July 2025) will maximise knowledge mobilisation by hosting a policy–clinical think tank event to identify barriers and facilitators to incorporating research into practice (including those identified in workstream 1) and integrate learning from workstreams 2 and 3 into innovative clinical work.

### Workstream 1: coming together – ethics and collaborative working event

**Fig. 2:**
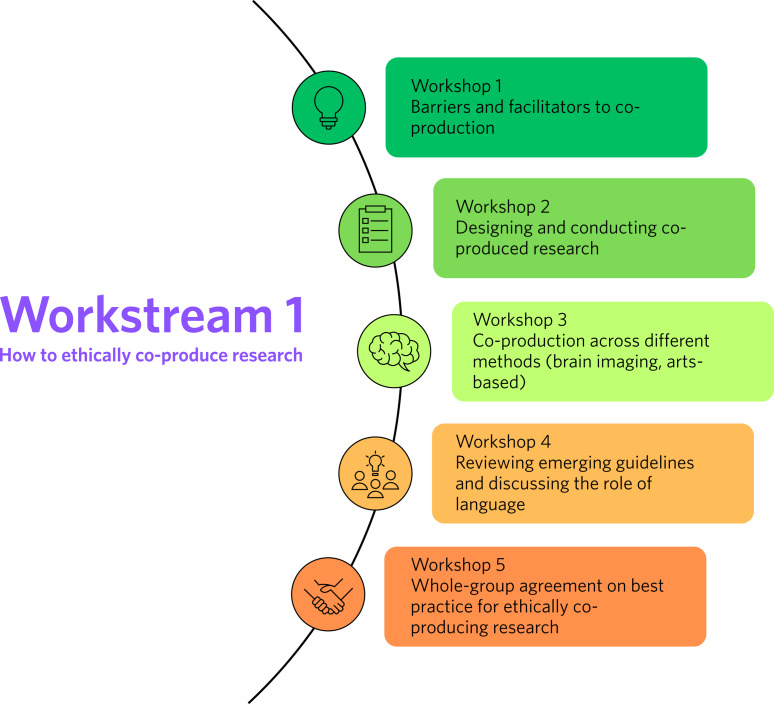
Workstream 1 co-production workshops.

Workstream 1 will consist of a series of five interdisciplinary workshops with Autistic individuals who have living/lived experience of eating disorders, researchers and clinicians to create guidelines on the ethical co-production of research in this field. A parallel process will take place to support the development of co-produced guidance for individuals with intellectual disabilities, a neglected population in the field of eating disorders that requires expansion.^25,26^ The five workshops (Fig. 2) will reflect on barriers to and facilitators of co-production, before guiding participants through the research process to identify associated aspects requiring ethical considerations at each stage. This will include topics associated with differing research methodologies such as brain imaging, experimental psychology and arts-based methodologies. The co-produced guidance, including agreement on use of language, will inform the principles that will underpin EDAC, its workstreams and any collaborative research aligned with the network.

### Workstream 2: setting the agenda – arts-based priority setting

Workstream 2 will recruit 55 Autistic people with living/lived experience of eating disorders. Arts-based methodologies will be used to capture perspectives and experiences of diverse and underrepresented groups including adults with intellectual disabilities, men, LGBTQ+ groups, minoritised ethnic groups and the full range of eating disorder presentations (with previous research having primarily focused on anorexia nervosa). Participants will also take part in a priority-setting exercise, identifying key areas that require further research. The priority-setting activity will be carried out based on the James Lind Alliance framework (https://www.jla.nihr.ac.uk/jla-guidebook/). This involves working with Autistic people through the following stages: (a) gathering evidence uncertainties (i.e. research gaps identified in workshop 1); (b) summarising responses gathered; (c) evidence-checking (checking this against existing research gaps); (d) interim priority setting (summarising questions to be discussed during workshop 2); (e) workshopping (taking the highest-ranked questions and agreeing a top-ten list of priorities). This workstream will primarily use photovoice, an accessible qualitative and participatory methodology which allows researchers to gain a better understanding of the living/lived experiences of people from underrepresented and underserved populations.^27^ Participants are asked to take images that capture their experiences to share and discuss with the research team, supporting a shared narrative. This technique been used to empower community members to document and share their stories^28,29^ and has been shown to be an effective method for exploring the living/lived experience of young people with disabilities^30^ and neurodivergent individuals.^31^ On this occasion, the images and narrative will be qualitatively analysed to document the experiences of individuals with eating disorders, to set research priorities, and the photographs themselves will form part of an exhibition, raising awareness of the needs and issues of this community.

### Workstream 3: testing the approach – interdisciplinary networking/sandpit events

Workstream 3 will be a series of interdisciplinary, proof-of-concept studies aimed at supporting collaborative working and tackling the research priorities set by Autistic individuals with living/lived experience of eating disorders in workstream 2. This work will provide a space to test out innovative cross-disciplinary approaches. Owing to the diversity of the interdisciplinary network, this will include a range of methodologies with the potential to include (individually or in combination) neuroimaging, experimental psychology, arts-based methodologies, merging of existing data-sets between research groups and collaborative work on additional systematic reviews in the field. Applications from researchers proposing a specific proof-of-concept study will be reviewed and shortlisted by trained Autistic individuals with living/lived experience of eating disorders. Projects will be assessed based on adapted MRC assessment criteria, which include ratings of research importance, potential and resources requested. It is hoped that outcomes will include: (a) bridging of the eating disorder and autism research communities, collaborating on research addressing priorities set by Autistic individuals with living/lived experience; (b) proof-of-concept or pilot studies that will inform larger grant proposals; (c) connections and networks between multiple sites carrying out research in autism and eating disorders; and (d) development opportunities for early career researchers, including Autistic peer researchers (individuals who use their understanding of an topic to help generate information about their peers for research purposes).

### Workstream 4: knowledge mobilisation – policy and clinical partnerships

To maximise knowledge mobilisation and future pathways to have an impact in this field, we will host a policy–clinical think tank event. This will involve two workshops with clinicians, clinical service managers and policy makers to discuss barriers and facilitators to incorporating neurodiversity-affirming research into policy and clinical practice. Workstream 4 will also include implementation of PEACE^15^ in two Scottish eating disorder services, diversifying from its initial primary focus on adult eating disorder in-patient environments. This will include the co-production of a pragmatic assessment toolkit for eating disorders in Autistic individuals, incorporating research findings from prior workstreams and the extended literature directly into clinical practice.

### Employability opportunities and skills

A significant objective of this project is to not only support capacity-building but also to provide career development and employability skills to the Autistic community with lived experiences of eating disorders. This is an important objective of the project, as despite strengths and advantages that Autistic people can bring to the workforce, they remain significantly underemployed.^32^ We therefore aim to provide employability skills training for Autistic individuals, including development of research skills, with the potential to attract individuals with living/lived experience into academic careers. These opportunities are embedded throughout the workstreams as part of either the workstream activity or the related output. This includes peer researcher opportunities, interviewing, videoing and editing, website content creation, and reviewing research.

## Conclusions

The application of a neurodivergence lens to eating disorder research is fundamental to improving clinical outcomes for the up to 30% of individuals with eating disorders who display Autistic characteristics. This can only be achieved by meaningful co-production of research and actively exploring Autistic individuals’ experiences of eating disorders using accessible methodologies. As well as supporting novel interdisciplinary collaborations in eating disorder research and increased capacity in the field, EDAC fundamentally will be led by the priorities of the Autistic community and underrepresented populations. Ultimately, the aim of EDAC is to provide a platform for innovative collaborations and to embed a neurodiversity-affirming culture within eating disorder research. This will support a new generation of researchers in the development of more inclusive, meaningful and translatable research that has the potential to significantly improve clinical outcomes.

## Data Availability

Data availability is not applicable to this article as no new data were created or analysed in its preparation.

## About the authors

**Fiona Duffy** is a consultant clinical psychologist at NHS Lothian Child and Adolescent Mental Health Services, Edinburgh, UK and a senior lecturer at the School of Health in Social Science, University of Edinburgh, Edinburgh, UK. **Karri Gillespie-Smith** is a senior lecturer at the School of Health in Social Science, University of Edinburgh, Edinburgh, UK. **Helen Sharpe** is a senior lecturer at the School of Health in Social Science, University of Edinburgh, Edinburgh, UK. **Kyle Buchan** is a research assistant at the School of Health in Social Science, University of Edinburgh, Edinburgh, UK. **Emy Nimbley** is a post-doctoral researcher at the School of Health in Social Science, University of Edinburgh, Edinburgh, UK. **Ellen Maloney** is a peer researcher at the School of Health in Social Science, University of Edinburgh, Edinburgh, UK. **Michelle Sader** is a post-doctoral researcher at the Aberdeen Biomedical Imaging Centre, University of Aberdeen, Aberdeen, UK; **Sarah Kettley** is a professor at Material and Design Innovation at Edinburgh College of Art, Edinburgh, UK; **Jess Kerr-Gaffney** is a post-doctoral researcher at the Institute of Psychiatry, Psychology and Neuroscience, King's College London, London, UK. **Gordon Waiter** is Reader in Medical Imaging and Director of the Aberdeen Biomedical Imaging Centre, University of Aberdeen, Aberdeen, UK. **Kate Tchanturia** is a professor at the Department of Psychological Medicine, Institute of Psychiatry, Psychology and Neuroscience, King's College London, London, UK and a consultant psychologist at National Eating Disorders Service, South London and Maudsley National Health Service (NHS) Foundation Trust, London, UK.
